# Surgical Outcomes and Exploratory Preoperative Risk Stratification for Invasive Pulmonary Fungal Infections in Paediatric Patients: A Single‐Center Retrospective Study

**DOI:** 10.1111/myc.70201

**Published:** 2026-06-18

**Authors:** Gang Yang, Chang Xu, Taozhen He, Xiao‐xi Lu, Xia Guo, Miao Yuan, Yanan Li

**Affiliations:** ^1^ Department of Pediatric Surgery, West China Hospital Sichuan University Chengdu Sichuan China; ^2^ Department of Pediatrics, West China Second University Hospital Sichuan University Chengdu China

**Keywords:** immunocompromised, invasive pulmonary fungal infection, lung resection, paediatric, preoperative risk stratification

## Abstract

**Background:**

Invasive pulmonary fungal infections (IPFIs) in immunocompromised children carry high morbidity and may interrupt life‐saving chemotherapy or haematopoietic stem cell transplantation (HSCT). Evidence on perioperative risk stratification for surgical management is limited.

**Patients and Methods:**

We conducted a single‐center retrospective cohort study of patients < 16 years with proven/probable IPFI who underwent lung resection from 2017 to 2025, with fungal infection confirmed by pathology. Preoperative clinical variables, laboratory tests and CT features were collected. Outcomes included intraoperative blood transfusion, severe postoperative complications, 90‐day mortality and time to resumption of chemotherapy/HSCT. An exploratory preoperative weighted risk score was derived and evaluated.

**Results:**

Thirty‐three patients were included (median age 9 years; 75.8% male). *Aspergillus* was the most common pathogen (72.7%); 57.6% had centrally located lesions and 12.1% had multilobe involvement. Procedures were mainly lobectomy (66.7%) or wedge resection (30.3%), with VATS in 45.5%. Peripheral lesion location was associated with less intraoperative transfusion, while hemoptysis and central lesion predicted delayed resumption of chemotherapy/HSCT; preoperative platelet count < 100 × 10^9^/L, multilobe involvement and central location were significantly associated with severe complications. Integrating these independent findings, a 0–8 point composite risk score was constructed (optimism‐adjusted C‐index: 0.948). The model effectively stratified outcomes: severe complications occurred in 0% (low), 26.7% (moderate) and 75% (high) (*p* = 0.004). Among survivors, median time to resume chemotherapy/HSCT was 18 days (low) versus 35 days (moderate) versus 78 days (high) (*p* < 0.001). All 90‐day deaths occurred in the high‐risk group (*p* < 0.001).

**Conclusions:**

Surgery can support source control and timely oncologic treatment in selected paediatric IPFI. A simple, statistically validated preoperative score may help anticipate surgical risk and recovery time and identify a high‐risk subgroup with poor short‐term outcomes.

## 1 Introduction

Invasive pulmonary fungal infections (IPFIs) pose a severe threat to paediatric patients with underlying haematological malignancies or immunosuppression [1, 2, 3]. These infections are associated with high morbidity and mortality, primarily due to vascular invasion leading to complications like hemoptysis or dissemination [4]. While systemic antifungal therapy forms the primary treatment, its response is limited due to poor drug penetration. Surgical resection, including wedge resection and lobectomy, represents an effective therapeutic strategy for localized pulmonary fungal infections, demonstrating significant efficacy in achieving source control and facilitating timely resumption of subsequent chemotherapy or haematopoietic stem cell transplantation (HSCT) in appropriately selected patients [5, 6].

Despite these insights, the existing literature is constrained by significant heterogeneity in study populations and the absence of risk‐stratified analyses, which limits the generalizability and clinical applicability of the findings. Furthermore, the differential efficacy and risks of surgical intervention across patients with varying disease severity remain poorly characterized, and no risk stratification methods currently exist to guide perioperative decision‐making or outcome prediction in this population.

This single‐center retrospective study aims to assess surgical outcomes in paediatric patients with pulmonary IPFI who underwent surgery, and to establish a preoperative risk‐stratification framework. By categorizing patients based on relevant clinical characteristics, the study seeks to evaluate differences in surgical safety and efficacy. The findings are expected to contribute to the optimisation of multidisciplinary management in immunocompromised children, with the potential to reduce mortality and facilitate earlier resumption of oncologic therapy.

## 2 Patients and Methods

This study was a single‐center retrospective cohort study, which had been approved by the Ethics Committee of West China Hospital (Ethics Approval No.: 2024110). The study protocol complied with the Declaration of Helsinki and relevant ethical standards. Due to the retrospective nature of this study, all data of the included patients were de‐identified, so the informed consent was waived.

### 2.1 Inclusion and Exclusion Criteria

Patients diagnosed and any surgical procedures occurred between January 1, 2017, and August 31, 2025 were included if they met the following criteria: (1) Paediatric patients under 16 years of age at the time of diagnosis with invasive pulmonary fungal infection; (2) Cases classified as proven or probable IPFI according to the EORTC/MSG criteria [7, 8, 9]; (3) Patients with an immunocompromised state due to chemotherapy for haematological malignancies or solid tumours; (4) Patients who underwent lung surgery (e.g., wedge resection, segmentectomy, or lobectomy) for reasons such as antifungal treatment failure, hemoptysis, or pre‐HSCT clearance; (5) Fungal infection confirmed by pathological exam. Patients were excluded if the surgery was performed for biopsy or if essential records for analysis were missing, such as CT scans, surgical reports, or follow‐up < 3 months. Patients with disseminated fungal infection were also included if thoracic surgery was performed for the pulmonary fungal infection.

### 2.2 Surgical Indications and Fitness Assessment

The indication for surgery was established through a multidisciplinary team consensus involving paediatric haematologists, thoracic surgeons and infectious disease specialists. Indications included failure of conservative antifungal therapy, massive hemoptysis, or the need to clear residual lesions prior to planned HSCT. Complete pulmonary function tests (FEV1, DLCO) were frequently unfeasible due to the young age and poor cooperation of the critically ill children. Consequently, surgical fitness and cardiopulmonary reserve were evaluated based on clinical performance status, echocardiography and anesthesiology assessments. Patients presenting with uncorrectable severe coagulopathy or multi‐organ failure were deemed inoperable.

### 2.3 Data Extraction

For each patient, the electronic medical record was reviewed for demographic characteristics (age, sex), underlying conditions (type of haematological malignancy or immunosuppression, HSCT status), hemoptysis, the last preoperative laboratory exam, radiological features, surgical details (indication, type [e.g., wedge resection, lobectomy], approach [VATS or open], date), perioperative outcomes (complications per Clavien‐Dindo classification, 90‐day mortality); follow‐up data (survival status, time to resumption of chemotherapy/HSCT).

### 2.4 Radiological Classification

**FIGURE 1:**
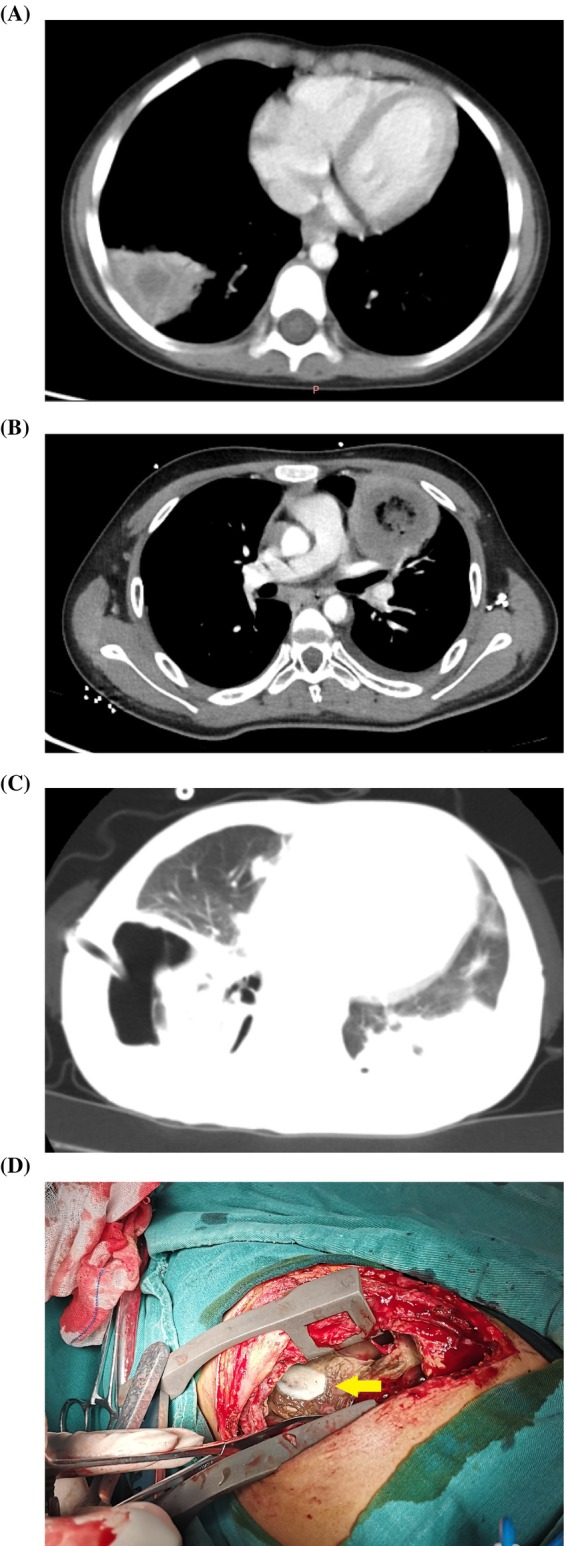
Radiological classifications and intraoperative findings of pulmonary fungal infection. Axial CT scans illustrating distinct morphological types: (A) 5‐year‐old girl with L1‐type acute lymphoblastic leukaemia and aspergillosis, peripheral location, nodule/mass type; (B) 13‐year‐old male with very severe aplastic anaemia and pulmonary mucormycosis, central location, cavity type; (C) 9‐year‐old male with T‐cell acute lymphoblastic leukaemia (L2 subtype, HOX11 gene positive) and mucormycosis, central location, diffuse/destroyed type. (D) Intraoperative photograph of the patient in Panel C, a disseminated, multilobe destroyed lung with visible extensive growth of fungal hyphae (arrow).

Based on preoperative CT morphology, lesions were categorized into three types: Solid Nodule/Mass Type: Well‐circumscribed nodules or masses without cavitation, representing localized infection. Cavitary Type: Lesions exhibiting cavities, halo signs, or air crescent signs, indicative of tissue necrosis secondary to angioinvasion and imply a potential bleeding risk. Diffuse/Infiltrative Type: Extensive consolidation or ‘destroyed lung’, representing severe disease burden and often involving multiple lobes. Lesion location was classified based on the proximity to hilar structures: Central type was defined as lesions located in the inner one‐third of the lung field or those directly invading/abutting the pulmonary hilum, main bronchus, or lobar vessels. Peripheral Type was defined as lesions located in the outer two‐thirds of the lung parenchyma, distant from the hilar structures. (Figure 1).

### 2.5 Antifungal Therapy Protocol

All patients received targeted systemic antifungal therapy preoperatively for a median of 3 weeks (primarily Voriconazole for *Aspergillus* and Liposomal Amphotericin B for Mucormycosis). Postoperatively, systemic therapy was continued based on MDT recommendations for a minimum of 6–12 weeks.

### 2.6 Outcomes

The outcomes of this study were designed to comprehensively evaluate the efficacy and safety of surgical intervention. Specifically, intraoperative blood transfusion was quantified as an indicator of the extent of angioinvasion and the associated technical complexity of the procedure. Postoperative complications were defined according to the Clavien‐Dindo classification; only severe complications (grade III–V) were extracted and analysed [10]. Postoperative 90‐day mortality was assessed as a surrogate for successful microbiological source control, because patients died more than 3 months after surgery most often due to relapse or progression of their underlying primary disease [11]. Additionally, the time from surgery to the resumption of either chemotherapy or haematopoietic stem cell transplantation (HSCT) was analysed to determine the impact of surgical management on the continuity of definitive oncologic therapy.

### 2.7 Exploratory Risk Stratification

To facilitate clinical decision‐making, we developed an exploratory risk stratification model aimed at predicting overall surgical outcomes, synthesizing the results from the independent outcome analyses. Specifically, the model was designed to encompass both surgical safety (severe complications) and oncologic recovery (time to resume therapy). Variables that demonstrated independent prognostic value in the multivariate analyses of these specific outcome domains were selected as candidate predictors.

To assign weights objectively while maintaining clinical applicability, integer points were allocated to each selected variable. This weighting process was primarily mathematically informed by the relative magnitude of their regression coefficients derived from the models, while simultaneously incorporating practical clinical considerations, such as the perioperative modifiability of specific risk factors (e.g., platelet transfusion). An overall risk score was then calculated for each patient by summing these points. The cohort was subsequently stratified into three tentative risk tiers—Low, Moderate and High—based on the distribution of cumulative scores.

### 2.8 Statistical Analysis

Descriptive statistics were used to summarize patient characteristics. Group comparisons were performed using ANOVA, Kruskal–Wallis, Chi‐square, or Fisher's exact tests, as appropriate. Multivariable modelling strategies were strictly tailored to the data type and event frequency of each outcome. First, for continuous outcomes (intraoperative blood transfusion and time to resume therapy), variables with *p* < 0.2 in univariate screening were entered into standard multiple linear regression. Second, for predicting severe complications, a rare event (*n* = 7), variables were evaluated using Firth's penalized logistic regression to avoid data separation bias. Third, for 90‐day mortality (*n* = 3), multivariable regression was not performed due to the critically low event count.

The exploratory risk score weights were derived from the Firth's regression coefficients. To evaluate model robustness and mitigate overfitting, internal validation was performed using 1000 bootstrap resamples to calculate the optimism‐adjusted C‐index. The probability of resuming therapy was evaluated via Kaplan–Meier curves and Log‐rank testing. A *p*‐value < 0.05 was considered significant. All analyses were performed using R version 4.5.2.

## 3 Results

### 3.1 Characteristics of Included Patients

A total of 33 patients were included in this study (Table 1). The cohort comprised 25 males (75.8%) with a median age of 9 years (IQR: 5–13 years). Regarding underlying hematologic/oncologic diseases, acute lymphoblastic leukaemia was the most common diagnosis (*n* = 17, 51.5%), followed by lymphoma (*n* = 7, 21.2%), acute myeloid leukaemia (*n* = 4, 12.1%), acute monocytic leukaemia (*n* = 2, 6.1%), very severe aplastic anaemia (*n* = 2, 6.1%) and pineal germinoma (*n* = 1, 3.0%). Hemoptysis was present in 4 patients (12.1%).

**TABLE 1:** Baseline characteristics and perioperative outcomes of included patients.

Characteristic	*N* = 33
Sex, *n* (%)	
Female	8 (24.2%)
Male	25 (75.8%)
Age, years, median (IQR)	9.0 (5.0–13.0)
Underlying disease, *n* (%)	
Acute lymphoblastic leukaemia	17 (51.5%)
Lymphoma	7 (21.2%)
Acute myeloid leukaemia	4 (12.1%)
Acute monocytic leukaemia	2 (6.1%)
Very severe aplastic anaemia	2 (6.1%)
Pineal germinoma	1 (3.0%)
Hemoptysis, *n* (%)	4 (12.1%)
Lesion Lobe, *n* (%)	
Right upper	2 (6.1%)
Right middle	1 (3.0%)
Right lower	9 (27.3%)
Left upper	7 (21.2%)
Left lower	10 (30.3%)
Multilobe	4 (12.1%)
CT Appearance, *n* (%)	
Nodule/Mass	13 (39.4%)
Cavity	13 (39.4%)
Diffuse/Destroyed type	7 (21.2%)
Lesion location, *n* (%)	
Central	19 (57.6%)
Peripheral	14 (42.4%)
Pathogen, *n* (%)	
*Aspergillus*	24 (72.7%)
*Mucor*	8 (24.2%)
*Cryptococcus*	1 (3.0%)
Haemoglobin, g/L, median (IQR)	102 (93–111)
Platelets, ×10^9^/L, median (IQR)	176 (98–285)
WBC, ×10^9^/L, median (IQR)	5.5 (3.8–9.3)
Surgical approach, *n* (%)	
Open	18 (54.5%)
VATS	15 (45.5%)
Type of resection, *n* (%)	
Wedge	10 (30.3%)
Lobectomy	22 (66.7%)
Pneumonectomy	1 (3.0%)
Intraoperative blood transfusion, mL, median (min—max)	200 (0–800)
Severe complications, *n* (%)	7 (21.2%)
Time to resume chemotherapy/HSCT, days, median (IQR)^a^	32 (19–56)
90‐day mortality, *n* (%)	3 (9.1%)

^a^ Data available for 31 patients (2 patients were not receiving chemotherapy/HSCT after surgery).

The pulmonary fungus infection lesions were in single lobe in 29 patients (87.9%) and multiple lobes in 4 patients (12.1%). The computed tomography (CT) appearances were nodule/mass (*n* = 13, 39.4%), cavity (*n* = 13, 39.4%) and diffuse/destroyed (*n* = 7, 21.2%). Lesions were centrally located in 19 patients (57.6%) and peripherally in 14 patients (42.4%). At the time of surgery, 31 patients had isolated pulmonary disease. Only 2 patients had suspected controlled extrapulmonary dissemination (one with sinus involvement, one with hepatic/sub diaphragm abscesses), but underwent lung resection to control the predominant life‐threatening pulmonary lesions.

Microbiologically, *Aspergillus* was the most frequently identified pathogen (*n* = 24, 72.7%), followed by *Mucor* (*n* = 8, 24.2%) and *Cryptococcus* (*n* = 1, 3.0%). Postoperative cultures and pathological examinations predominantly confirmed the preoperative presumed pathogens, and significant postoperative changes to the antifungal regimen due to unexpected resistance were not observed.

Surgically, a video‐assisted thoracoscopic surgery (VATS) approach was used in 15 patients (45.5%), while an open thoracotomy was performed in 18 patients (54.5%). The procedures included wedge resection in 10 cases (30.3%), lobectomy in 22 cases (66.7%) and pneumonectomy in 1 case (3.0%).

### 3.2 Intraoperative Blood Transfusion

Eighteen patients received intraoperative blood transfusion ranging from 200 to 800 mL. Six variables meeting the screening criteria (*p* < 0.2) were included in the multiple linear regression model (Table S1). Peripheral lesions were independently associated with significantly reduced intraoperative blood transfusion (mean reduction: 235.55 mL; 95% CI: −397.59 to −73.51; *p* = 0.012) (Table 2).

**TABLE 2:** Multivariate regression analysis of independent predictors for clinical outcomes.

Outcomes/Variables	Coefficient (*β*)	95% CI	*p*
**Intraoperative blood transfusion**	**(Linear regression)**		
Lesion location			
Central	Reference	—	—
Peripheral	−235.55	−397.59 to −73.51	0.012
**Severe complications**	**(Firth's penalized logistic regression)**		
Central location	2.99	0.06 to 8.13	0.045
Multilobe	3.61	0.88 to 8.53	0.007
Platelet < 100 × 10^9^/L	3.89	1.21 to 8.85	0.002
**Time to resume chemotherapy/HSCT**	**(Linear regression)**		
Hemoptysis	28.18	6.47 to 49.90	0.017
Lesion location			
Central	Reference	—	—
Peripheral	−27.01	−40.19 to −13.83	< 0.001

### 3.3 Postoperative Severe Complications

Severe complications occurred in 7 patients (21.2%). These complications included six Clavien‐Dindo grade III (insertion of chest tube for pneumothorax and pleural effusion in five, bronchoscopy with suctioning and lavage under general anaesthesia for persistent atelectasis in one) and one grade IV (kidney dysfunction).

The multivariate Firth's penalized logistic regression was conducted (Table 2). The analysis revealed that central lesion location (Coefficient = 2.99, *p* = 0.045), multilobe involvement (Coefficient = 3.61, *p* = 0.007), and low preoperative platelet count (Coefficient = 3.89, *p* = 0.002) were significantly associated with an increased risk of severe complications.

### 3.4 Time to Resume Chemotherapy/HSCT

The median time to resume therapy among survivors was 32 days. In the multiple linear regression model, the presence of preoperative hemoptysis was a strong predictor of delay, adding 28.18 days to the recovery time (*p* = 0.017). Conversely, a peripheral lesion location was associated with a significantly shorter delay (−27.01 days; *p* < 0.001) compared to central lesions (Table 2).

### 3.5 Mortality in 90 Days Postoperatively

Three deaths occurred within 90 days of surgery (9.1%). Due to the critically limited number of events, multivariable regression was unfeasible and not performed. Notably, all three fatal cases presented with multilobe lesions (accounting for 75% of patients with multilobe involvement) and developed severe postoperative complications (Clavien‐Dindo grade III or higher).

### 3.6 Risk Stratification Model and Internal Validation

A comprehensive risk score was constructed to reflect the multi‐domain challenges of IPFI surgery, integrating the significant findings from the independent outcome analyses. Based on the multivariate models (Table 2), four variables were selected due to their independent impact on either surgical safety or oncologic recovery: central lesion location, multilobe involvement, preoperative platelet count < 100 × 10^9^/L, and hemoptysis.

The point allocations were mathematically informed by the Firth's regression coefficients for severe complications. Central location (coefficient: 2.99) and multilobe involvement (coefficient: 3.61) justified weights of 3 points each. Hemoptysis, which independently predicted delayed chemotherapy resumption in the linear regression model, was assigned 1 point. Although preoperative thrombocytopenia demonstrated a high coefficient (3.89) for complications, it was conservatively assigned 1 point to reflect its partial perioperative modifiability through proactive platelet transfusion (Table S2).

Patients were stratified into low‐risk (0–2 points, *n* = 14, 42.4%), moderate‐risk (3–5 points, *n* = 15, 45.5%) and high‐risk (≥ 6 points, *n* = 4, 12.1%) categories (Table 3). Crucially, to evaluate the discriminative performance and mitigate overfitting, internal validation using 1000 bootstrap resamples was performed. The model demonstrated outstanding robustness, yielding an optimism‐adjusted C‐index of 0.948 (original C‐index: 0.957).

**TABLE 3:** Patient outcomes stratified by risk group.

Variable	Low risk, *n* = 14	Moderate risk, *n* = 15	High risk, *n* = 4	*p*
Intraoperative blood transfusion (mL), median (IQR)	0 (0–200)	200 (0–200)	200 (200–300)	0.048^a^
Severe complications, *n* (%)	0 (0%)	4 (27%)	3 (75%)	0.004^b^
Time to resume chemotherapy/HSCT (days), Median (IQR)	18 (12–21)	35 (32–75)	78	< 0.001^c^
90‐day mortality, *n* (%)	0 (0%)	0 (0%)	3 (75%)	< 0.001^b^

^a^ Kruskal–Wallis rank sum test.

^b^ Fisher's exact test.

^c^ Log‐rank test.

**FIGURE 2:**
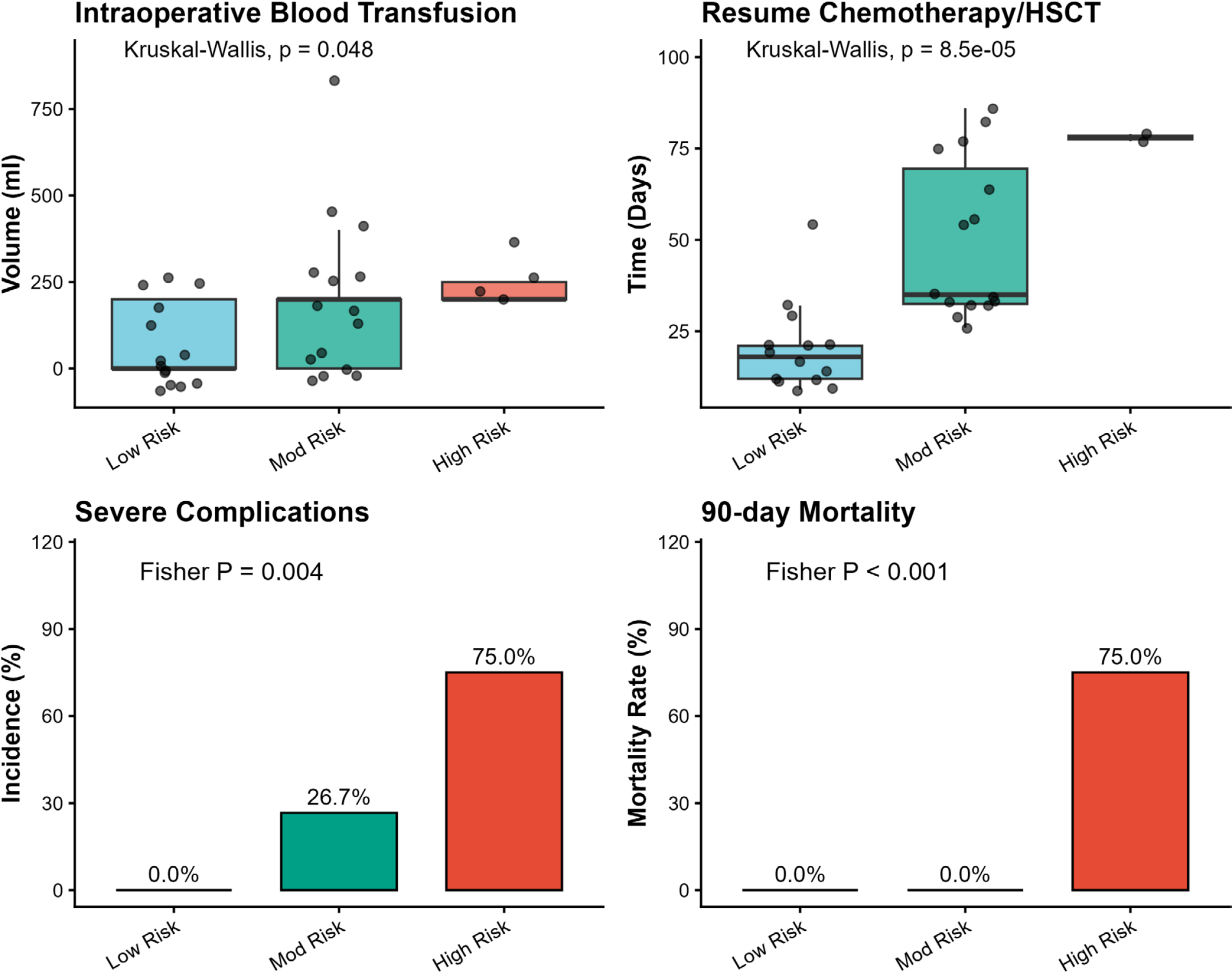
Discriminatory performance of the exploratory preoperative risk score across outcomes in paediatric invasive pulmonary fungal infection.

**FIGURE 3:**
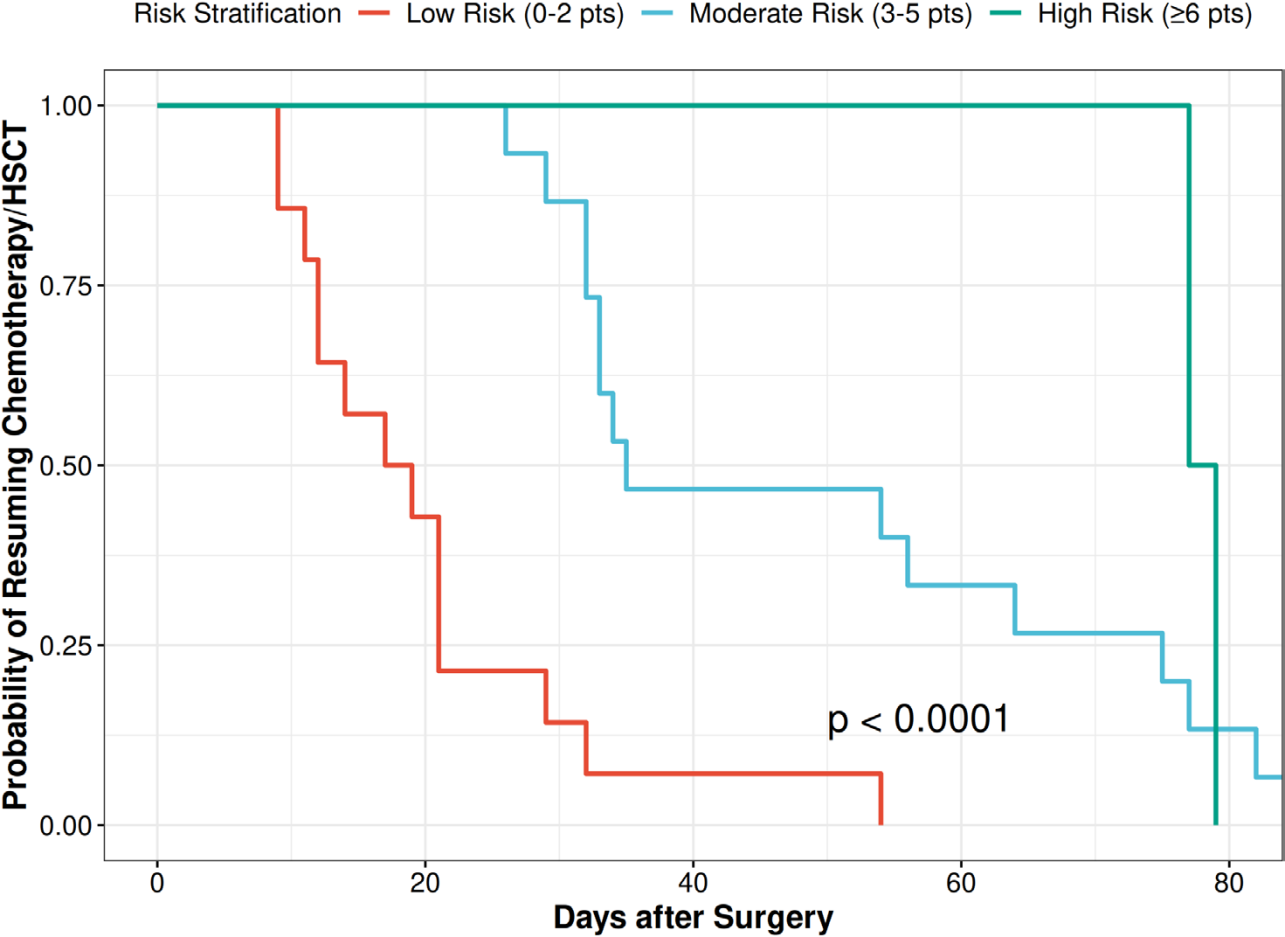
Kaplan–Meier survival curves illustrating the cumulative probability of resuming chemotherapy/HSCT stratified by preoperative risk groups.

The composite model effectively stratified outcomes across all clinical domains (Figure 2). Intraoperative blood transfusion was significantly higher in the high‐risk group (Table 3). The incidence of severe complications increased progressively with risk level (0% vs. 26.7% vs. 75%; *p* = 0.004). Regarding oncologic recovery, Kaplan–Meier analysis (Figure 3) demonstrated a highly significant difference in the probability of resuming chemotherapy/HSCT (Log‐rank *p* < 0.001). The low‐risk group achieved rapid resumption of therapy (median: 18 days), whereas the moderate‐risk (35 days) and surviving high‐risk patients (78 days) experienced substantial delays. Furthermore, all 90‐day deaths occurred exclusively in the high‐risk group (*p* < 0.001).

## 4 Discussion

Our study reaffirmed the utility of surgical resection as a salvage strategy for paediatric IPFIs with immunocompromised status. More importantly, we introduced a novel preoperative risk stratification model. By integrating critical symptom, laboratory exam, anatomical location and extent of the lesion, this model provides a granular framework to predict surgical complexity, safety and the crucial timeline for resuming oncologic therapy.

Because most IPFI occurs in patients with underlying malignant disease, the primary challenge in treatment is not merely eradicating the fungus, but to enable patients to resume chemotherapy or proceed to haematopoietic stem cell transplantation (HSCT). Our cohort demonstrated a median time to resumption of chemotherapy of 32 days, a finding that aligns with the results reported by Fati et al. and Hassan et al. [5, 12] Specifically, we observed that patients with peripheral lesions resumed chemotherapy significantly earlier (median difference of 27 days) compared to those with central lesions. This finding has profound clinical implications: for peripheral nodules, prolonged antifungal monotherapy may be inferior to early surgical intervention, as the latter allows for a rapid return to the primary life‐saving oncologic treatment.

Our analysis identified lesion location as the dominant predictor of surgical complexity. Central lesions were associated with significantly higher blood loss (reduction of 236 mL in peripheral cases). This correlates with the anatomical reality that central lesions often adhere to or invade the pulmonary hilum and major vascular structures, necessitating technically challenging dissections. Therefore, the procedures for central location lesion carry an inherent risk of vascular injury, requiring experienced thoracic surgical teams [13, 14].

Hemoptysis and thrombocytopenia are critical markers of poor prognosis. Hemoptysis in IPFI is a direct manifestation of angioinvasion by hyphal elements, signalling a biologically aggressive disease state [14]. Our study found that hemoptysis was independently associated with a nearly one‐month delay in resuming chemotherapy. Similarly, low platelet counts often reflect severe bone marrow failure and poor systemic reserve, increasing the risk of postoperative complications. These findings suggest that patients presenting with hemoptysis and thrombocytopenia represent a biologically distinct, higher‐risk subgroup.

Although statistical significance was limited by sample size, the clinical pattern regarding mortality was unequivocal: all three fatal cases in our cohort presented with multilobe involvement. This aligns with the multicenter analysis by Burgos et al., which identified patients with disseminated disease had worse prognosis [6]. Multilobe disease likely represents a systemic failure of host defence and a fungal burden that exceeds the capacity of local source control. Consequently, we propose that multilobe involvement should serve as a significant ‘red flag’. In such cases, surgery may not alter the dismal natural history of the disease unless it is performed strictly for life‐saving symptom control (e.g., stopping massive bleeding) rather than with curative intent.

A key feature of this study is the exploratory risk stratification model. Building on earlier descriptive work and our data, we propose a preliminary 0‐to‐8‐point scale that converts selected clinical variables into tentative risk groups [15, 16]. Importantly, this model is a synthesis of distinct analytical endpoints, ensuring it captures both surgical complexity/safety and oncological recovery. Crucially, rather than relying solely on empirical clinical judgement, the score weights were mathematically derived using Firth's penalized logistic regression to provide robust statistical justification despite the small sample size. Our analysis demonstrated that the independent prognostic impacts of central lesion location (coefficient: 2.99) and multilobe involvement (coefficient: 3.61) were approximately three times larger than the baseline unit. Consequently, these severe anatomical features were weighted with 3 points each. Hemoptysis, representing a strong predictor for delayed recovery, was assigned 1 point. Additionally, a preoperative platelet count of < 100 × 10^9^/L was conservatively assigned 1 point; this captures the risk associated with compromised systemic physiological reserve while acknowledging its partial correctability through perioperative transfusion.

Based on our scale, the low‐risk group (0–2 points) comprises nearly half of our cohort (42.4%); these patients typically presented with single, peripheral lesions and adequate platelet counts. They achieved a 0% mortality rate, 0% severe complication rate, and resumed chemotherapy in a median of 18 days. For this group, we advocate for an aggressive, early surgical approach. The benefits clearly outweigh the risks, and early resection minimizes the delay in treating the underlying malignancy. Patients in the moderate‐risk group (3–5 points) often had central lesions or mild physiological derangements. While surgery remains viable, the complication rate rose to 26.7%, and the time to resume chemotherapy extended to 35 days. Therefore, surgery should be performed with enhanced perioperative preparation. This includes aggressive platelet transfusion thresholds, preoperative nutritional support, and the availability of advanced postoperative intensive care. The high‐risk group (≥ 6 points) represents the ‘surgical danger zone’. Characterized by central, multilobe disease combined with hemoptysis or thrombocytopenia, these patients faced a 75% 90‐day mortality rate in our study. In these scenarios, surgery should be reserved strictly as a measure of last resort for immediate life‐threatening emergencies, and families must be counselled regarding the extremely poor prognosis. Less‐invasive procedures such as percutaneous drainage or irrigation should be considered [17].

Several limitations must be acknowledged. First, the retrospective design introduces selection bias, as patients deemed too unstable for anaesthesia were excluded, potentially underestimating the true mortality of IPFI. Second, the relatively small sample size constitutes a notable methodological limitation. We fully acknowledge that evaluating a newly developed risk score solely on its derivation cohort introduces the risk of overfitting, effectively creating a circular logic or ‘self‐fulfilling prophecy’. To rigorously address this inherent bias, we performed internal validation using a 1000‐resample bootstrapping approach. The resulting optimism‐adjusted C‐index of 0.948 serves as compelling evidence that our model's discriminative validity is highly robust and not merely an artefact of the scoring definitions. Nonetheless, while bootstrapping effectively corrects for internal optimism, it cannot replace external validation. Prospective studies in independent, multi‐institutional cohorts remain imperative to confirm its generalizability before this tool can be widely adopted in clinical practice.

## 5 Conclusion

In conclusion, surgical resection is a vital component of the multidisciplinary management of paediatric IPFI, offering a pathway to resume essential oncologic therapy. However, due to the heterogeneity in severity of patients, we demonstrate that anatomical location, disease extent, and haematological status profoundly influence surgical outcomes. The proposed risk stratification model helps to guide clinical decision‐making. By identifying low‐risk candidates for early intervention and flagging high‐risk patients for caution, this framework aims to maximize the therapeutic benefit of surgery while minimizing preventable harm in this vulnerable paediatric population.

## Author Contributions

**Miao Yuan:** data curation, validation. **Xiao‐xi Lu:** data curation. **Taozhen He:** data curation. **Chang Xu:** conceptualization, methodology, investigation. **Gang Yang:** conceptualization, methodology, software, data curation, investigation, formal analysis, supervision, visualization, writing – original draft, writing – review and editing. **Yanan Li:** data curation, methodology. **Xia Guo:** data curation, validation.

## Funding

The authors have nothing to report.

## Ethics Statement

This study was a single‐center retrospective cohort study, which had been approved by the Ethics Committee of West China Hospital (Ethics Approval No.: 2024110).

## Conflicts of Interest

The authors declare no conflicts of interest.

## Supporting information


**Table S1:** Univariate analysis of factors associated with clinical outcomes (*p*‐value).
**Table S2:** The proposed preoperative risk stratification scoring system.

## Data Availability

The data that support the findings of this study are available on request from the corresponding author. The data are not publicly available due to privacy or ethical restrictions.
