# Supplementary material for: Surgical Outcomes and Exploratory Preoperative Risk Stratification for Invasive Pulmonary Fungal Infections in Paediatric Patients: A Single‐Center Retrospective Study
Source: Mycoses. 2026 Jun 18;69(6):e70201. doi: 10.1111/myc.70201 (PMC13277295; doi:10.1111/myc.70201)
Supplement: Supplementary file 1 — Table S1: Univariate analysis of factors associated with clinical outcomes (p‐value). Table S2: The proposed preoperative risk stratification scoring system. [file MYC-69-e70201-s001.docx]

Table S1. Univariate analysis of factors associated with clinical outcomes (*P*-value)

| **Variables** | **Intraoperative Blood Transfusion** | **Severe Complications** | **Time to Resume Chemotherapy/HSCT** | **90-day Mortality** |
| --- | --- | --- | --- | --- |
| Sex | 0.408 | 0.208 | 0.592 | 0.112 |
| Age | 0.262 | 0.499 | **0.037*** | 0.168 |
| Underlying disease | **0.106** | 0.998 | 0.571 | >0.999 |
| Hemoptysis | 0.957 | **0.159** | **0.002** | >0.999 |
| Lesion lobe | **0.014** | 0.683 | 0.566 | >0.999 |
| CT appearance | **0.013** | 0.883 | 0.223 | >0.999 |
| Lesion location | **0.034** | 0.995 | **<0.001** | >0.999 |
| Pathogen | 0.452 | 0.995 | 0.281 | >0.999 |
| Hb (g/L) | **0.061** | 0.755 | 0.884 | 0.975 |
| Platelet (×10⁹/L) | **0.147** | **0.081** | 0.452 | 0.616 |
| WBC (×10⁹/L) | 0.665 | 0.280 | 0.955 | 0.186 |

†Bold values indicate or meeting the screening criteria (*P* < 0.2) for multivariate entry. However, multivariate analysis for 90-day mortality was not performed due to the limited number of events (n=3), which precluded stable parameter estimation.

Table S2. The proposed preoperative risk stratification scoring system.

| **Risk factors** | **Score** |
| --- | --- |
| Central location | 3 |
| Multilobe involvement | 3 |
| Hemoptysis | 1 |
| Platelets < 100×10⁹/L | 1 |

†Sum of points: 0-2, low-risk; 3-5, moderate-risk; ≥ 6, high-risk
